# Machine learning nested in multiphysics finite element analysis: application to flash sintering

**DOI:** 10.1007/s10845-025-02725-y

**Published:** 2025-12-05

**Authors:** Ran He, Mingxuan Xia, Peter Polak, Baber Saleem, Xiaoxia Yu, Jingzhe Pan

**Affiliations:** School of Engineering, University of Leicester, Leicester, LE1 7RH UK

**Keywords:** Nested machine learning, Artificial neural network, Finite element analysis, Multiphysics, Digital twin

## Abstract

Finite element modelling is a powerful tool for predicting temperature field and deformation in flash sintering. However, conventional finite element modelling approaches often rely on empirical constitutive models, which struggle to capture the highly complex, nonlinear behaviour of materials under flash sintering conditions. To overcome this limitation, this study develops a framework that can train artificial neural networks (ANNs) when nested in multiphysics finite element analysis. By replacing empirical rules with ANNs, the finite element model can learn directly from data and, thus, operate as a digital twin of the flash sintering process. A two-step training strategy is adopted: pretraining of ANNs using synthetic data generated from empirical rules, followed by nested retraining within a commercial finite element package using experimental data. To enable the second-step training, a novel backpropagation algorithm integrated with the Adam optimiser is developed. The results demonstrate a significant improvement in predictive accuracy: the mean squared error between predicted and measured strains decreased from 0.0316 (modified Olevsky’s constitutive law) to 0.0034 (ANNs). Beyond improving model fidelity, the approach enables continual adaptation as new data become available and is extensible to all engineering problems governed by differential equations.

## Introduction

Finite Element (FE) analysis has long been a foundational tool in engineering simulations, enabling precise modelling and evaluation of complex systems. This technique has significantly enhanced our ability to predict system behaviour under various conditions. However, conventional FE analysis primarily provides prescriptive insights, often disconnected from the real-time operational states of physical systems. To bridge this gap, the concept of digital twin has emerged as a natural progression from FE analysis (Boschert & Rosen, 2016), integrating numerical simulation with real-time sensor data, advanced analytics and machine learning. Digital twins are virtual replicas that dynamically mirror the behaviour and state of their physical counterparts, allowing for continuous monitoring, real-time diagnostics and predictive decision-making. This evolution from FE models to dynamic digital twins represents a significant advancement in simulation capabilities, enabling more accurate, responsive and insightful analysis.

FE models typically consist of two distinct components: fundamental scientific principles, such as energy conservation, and empirical rules, such as constitutive laws. Whilst fundamental principles are inviolable, empirical rules often rely on observations and assumptions that may not adequately capture the complexities of real-world behaviour. Recently, machine learning techniques have been adopted to create surrogate models approximating FE solutions for improved computational efficiency (Kohar et al. 2021; A. Li et al. 2020; Sakaridis et al. 2022). However, these data-driven approaches commonly treat existing mathematical models as ground truth, a premise that can be problematic given the inherent limitations of empirical rules. Nevertheless, machine-learning pipelines have already demonstrated value in broader manufacturing contexts—for example, in forecasting complex industry-linked indices (Akyüz et al. 2024)—highlighting their potential when carefully integrated with physics-based models. This study proposes a modelling approach that retains fundamental scientific principles but replacing empirical rules with artificial neural networks (ANNs). The inherent adaptability of ANNs transforms conventional FE models into digital twins capable of continuously learning real-world system behaviours.

**Fig. 1:**
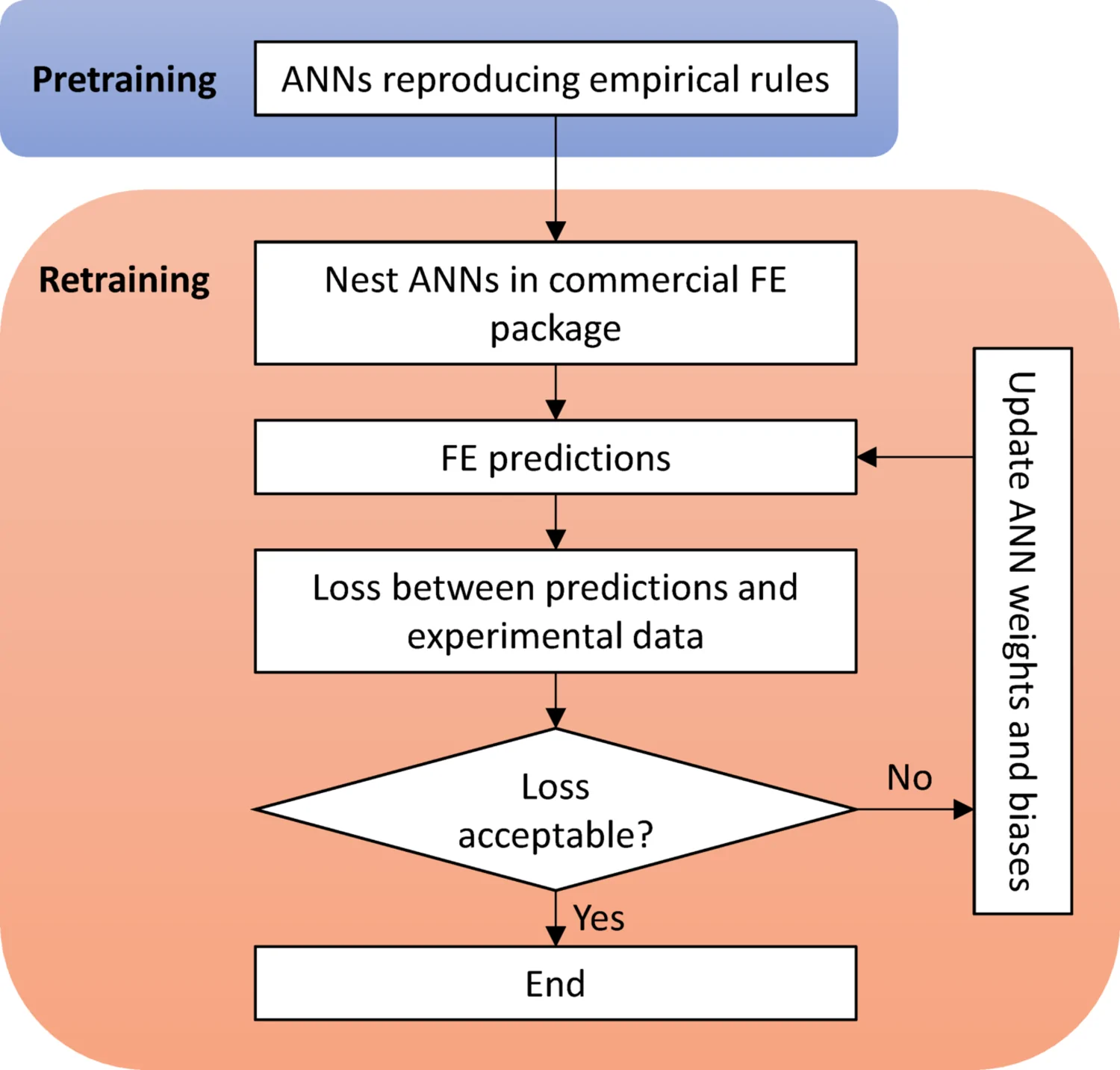
Overall workflow detailing approach proposed in this study

In our previous work (He et al., 2024), developing a digital twin for viscous deformation of a ceramic during conventional sintering was attempted by replacing the constitutive law with an ANN in the FE analysis. Whilst that approach successfully captured measured deformation during sintering, the ANN was trained externally using experimental data on small samples. For a true digital twin, however, needs to learn from actual manufacturing data, such as surface temperatures and global sintering deformations measured during the sintering process. The aim of this study is therefore to establish a framework for training ANNs nested in multiphysics FE analysis, with feasibility demonstrated using flash sintering. The present study introduces two major developments: (i) the training of ANNs while nested in a commercial FE package and (ii) the application of this ANN-nested modelling framework to a multiphysics process. Because the available experimental data were limited (Francis & Raj, 2012), a two-step training strategy was adopted to reduce data requirements (He et al., 2024; Linka et al., 2021). In the first step, the ANNs were pretrained to reproduce the empirical rules; in the second step, they were retrained using the experimental data. The overall workflow is summarised in Fig. 1.

A key challenge in nested training is computing the derivatives of the loss function with respect to ANN weights and biases, when the loss is defined in terms of measured variables rather than the ANN output variables. This requires calculating the derivatives of measured variables with respect to output variables. Several approaches were developed to enable nested training. SelfSim (also known as *autoprogressive training* or *self-learning simulation*) introduced by Ghaboussi et al. (Aquino and Brigham 2006; Gandomi and Yun 2015; Ghaboussi et al. 1998; Hashash et al. 2003; Youssef M. A. Hashash et al. 2006; Jung et al. 2007; Jung and Ghaboussi 2006; Shin and Pande 2000; Sidarta and Ghaboussi 1998; Yun et al. 2007; Gun Jin Yun et al. 2008, 2012) trains ANNs to capture complex stress-strain behaviours using global force-displacement data from structural testing. This approach, however, is limited to cases where ANN variables can be directly obtained and assumed to be true under the global loading in the simulations. Hybrid physics-informed neural network/ANN-physics models offer an alternative by replacing unknown or empirical terms in ordinary and partial differential equations (Machalek et al. 2021; Mitusch et al. 2021; Yucesan and Viana 2021). In such cases, the derivatives of measured variables with respect to output variables can be directly derived from the differential equations. Nonetheless, in many practical situations, this derivation is highly non-trivial or infeasible. For instance, in the work by Mohr and colleagues (Li et al. 2019, 2022, 2023; Pandya et al. 2020), the measured variable was the total axial force of dog-bone specimens $\:{F}_{lm}^{NN}$, whilst the ANN output variable was the deformation resistance term $\:{k}_{lmn}^{NN}$. To compute the partial derivative $\:\partial\:{F}_{lm}^{NN}/\partial\:{k}_{lmn}^{NN}$, the authors had to introduce assumptions and simplifications, limiting the generality of their approach to other engineering problems. In this study, we propose a generalised nested training approach by ignoring the absolute values of these derivatives. Its difference from the aforementioned existing approaches is presented in Table 1.

**Table 1:** Differences between proposed and existing approaches

Approach	Present study	SelfSim	Hybrid physics-informed neural networks/ ANN-physics models	Mohr and co-workers
Key idea	Replace empirical rules with ANNs nested in commercial FE	Train ANNs to reproduce material behaviour directly from global response data (e.g., force–displacement)	Replace unknown/empirical terms with ANNs nested in governing differential equations	Replace deformation resistance term with an ANN nested in commercial FE
Solution for derivatives of measured variables with respect to output variables	No requirement for any calculation of the derivatives, and, thus, universal to various problems	–	Derivatives are directly derived from differential equations	Derivatives are calculated with assumptions and simplifications
Applicability	All engineering problems governed by differential equations	ANN variables can be directly obtained and assumed to be true under global loading in simulations	Differential equations are explicitly available	Specific plastic model used in their studies

Flash sintering was selected as a demonstrative case in this study due to its multiphysics process and highly sophisticated deformation behaviour. As an advanced ceramic processing, flash sintering enables rapid densification by applying an electric field, where a significant portion of heat is generated internally through Joule heating (Yu et al., 2017). This internally generated heat allows for accelerated sintering at lower furnace temperatures and shorter times compared to conventional sintering. During sintering, the ceramic undergoes shrinkage as particles bond and pores close. This shrinkage is driven by the reduction of surface and interfacial energies, which promotes mass transport and leads to densification and volume contraction. Whilst the process is highly efficient, reducing energy consumption and production time, it is prone to issues like distortion of the material (Jha et al., 2016; Serrazina et al., 2022). Distortion occurs due to nonuniform heating and electric field distribution, causing nonuniform densification and stress concentration. This leads to dimensional inaccuracies and mechanical defects in the final product, limiting its application in precision ceramic manufacturing.

Most computational efforts in the literature focused on analysing temperature distributions in the samples during flash sintering without considering sintering deformation (Grasso et al., 2011; Mazo et al., 2023; Ni et al., 2022; Pereira Da Silva et al., 2015; Serrazina et al., 2019, 2022; Serrazina, Senos, Serrazina et al., 2020a, b; Serrazina, Vilarinho, Serrazina et al., 2020a, b; Xiao et al., 2023; Yoshida et al., 2018). A mathematical model was presented by Zhang et al. (2015) to predict the occurrence of thermal runaway, i.e., the onset temperature for flash sintering. Manière et al. (2022) proposed incorporating cumulative thermal energy at defects into the porosity calculation for flash sintering. Li et al. (2021) translated measured sample densities into uniform isotropic shrinkages in their FE models, whilst Arya et al. (2022) applied a master sintering approach to model densification. Only two studies (Koutoati et al., 2024; Wang et al., 2023) were found using constitutive laws to simulate the sample deformation during flash sintering. However, apparent discrepancies were observed between simulated and measured relative densities in Wang et al. (2023), and Koutoati et al. (2024) did not validate their simulation results against experimental data. Thus, there is a clear need for a computational approach capable of accurately predicting temperature field and deformation during flash sintering.

## Mathematical equations for flash sintering

The state-of-the-art mathematical models for flashing sintering, as reported in the literature, are presented in this section. The governing equations for the electric field are derived from Maxwell’s equations (Cheng, 1989). In a stationary coordinate system, the point form of Ohm’s law is expressed as

1 $$ {\mathbf{J}} = \Sigma {\mathbf{E}},$$

where $\:\mathbf{J}$ is the current density, $\:\varSigma\:$ is the electrical conductivity and $\:\mathbf{E}$ is the electric field strength. $\:\varSigma\:$ is modelled as a density-dependent weighted average, given by (Li et al., 2021)

2 $$ \Sigma = \Sigma _{g} \exp \left( {\frac{{ - Q_{g} }}{{RT}}} \right) + \left[ {\Sigma _{f} \exp \left( {\frac{{ - Q_{f} }}{{RT}}} \right) - \Sigma _{g} \exp \left( {\frac{{ - Q_{g} }}{{RT}}} \right)} \right]\frac{{\rho - \rho _{g} }}{{\rho _{f} - \rho _{g} }},$$

where superscripts * g* and * f* denote the green body and fully dense material, respectively, * Q* is the activation energy for electrical conductivity, * R* is the gas constant, * T* is the temperature and $\:\rho\:$ is the material density. The continuity equation is given by (Cheng, 1989)

3 $$ \nabla \cdot {\mathbf{J}} = 0.$$

The heat equation, which governs thermal behaviour, is given by (Karwa, 2020)

4 $$ \rho C_{p} \dot{T} + \nabla \cdot \left( {{\mathbf{q}} + {\mathbf{q}}_{{r}} + {\mathbf{q}}_{{c}} } \right) = Q_{e} ,$$

where $\:{C}_{p}$ is the specific heat capacity, $\:\dot{T}$ is the temperature rate, $\:\mathbf{q}$ is the heat flux vector by conduction, $\:{\mathbf{q}}_{\boldsymbol{r}}$ and $\:{\mathbf{q}}_{\boldsymbol{c}}$ are the heat flux vectors on the surface by radiation and convection, respectively, and $\:{Q}_{e}$ is the resistive (Joule) heating. $\:\mathbf{q}$ can be expressed as (Karwa, 2020)

5 $$ {\mathbf{q}} = - k\nabla T, $$

where * k* is the thermal conductivity. Schlichting et al. (2001) found that *k* was density-dependent and a Maxwell’s expression showed an adequate agreement with experimental measurement. Hence, we have

6 $$ k = k_{f} \frac{{2\rho }}{{3\rho _{f} - \rho }}.$$

The net inward heat flux from surface-to-ambient radiation is given by (Raj, 2012)

$$ - {\mathbf{n}} \cdot {\mathbf{q}}_{{\mathbf{r}}} = \in \beta \left( {T_{{amb}} ^{4} - T_{s} ^{4} } \right). $$

where $\:\mathbf{n}$ is the unit surface normal vector, * ∈ * is the surface emissivity, $\:\beta\:$ is the Stefan-Boltzmann constant, $\:{T}_{amb}$ is the ambient temperature and $\:{T}_{s}$ is the surface temperature of material. The convective heat flux from surface to ambient is described by (Karwa, 2020)

7 $$ - {\mathbf{n}} \cdot {\mathbf{q}}_{{\mathbf{c}}} = h\left( {T_{{amb}} - T_{s} } \right), $$

where * h* is the convective heat transfer coefficient for air. $\:{Q}_{e}$ is described by (Karwa, 2020)

8 $$ Q_{e} = {\mathbf{J}} \cdot {\mathbf{E}}. $$

The sintering deformation is assumed to follow the governing equations for viscous deformation in solid mechanics, employing Olevsky’s constitutive law (Van Nguyen et al., 2016). Full details of the constitutive law are provided in Appendix. This study found that a modification to Olevsky’s constitutive law was required to reasonably capture the deformation behaviour in flash sintering, Specifically, a material constant, $\:{C}_{s1}$, needs to be made dependent on the heating rate such that

9 $$ C_{{s1}} = C_{a} \exp \frac{{C_{b} }}{{\dot{T}}} + C_{c} .$$

where $\:{C}_{a}$, $\:{C}_{b}$ and $\:{C}_{c}$ are the material constants.

Equations (1)-(8) were adopted from previous studies, where they have been shown to be effective and widely used for flash sintering (Li et al., 2021; Pereira Da Silva et al., 2015), whilst further details are provided in Appendix to justify Eq. (9).

## A case study of finite element analysis showing unsatisfactory predictions

A flash-sinterforging experiment was conducted by Francis and Raj (2012) using nanograined 3 mol% yttria-stabilized zirconia (3YSZ). They provided measurements of surface temperature, input power and effective and volumetric strains. Such experimental data, covering both thermal and mechanical responses, are rare in the literature and were therefore selected as the basis for the present case study. Nine combinations of applied voltage and pressure were employed in their experiments: 100 V & 12 MPa, 100 V & 9 MPa, 100 V & 7.5 MPa, 100 V & 5 MPa, 100 V & 3 MPa, 100 V & 1.5 MPa, 200 V & 5 MPa, 50 V & 5 MPa and 0 V & 5 MPa. The last case (0 V & 5 MPa) was conventional sintering, whilst the remaining cases were flash sintering. Their specimens consisted of 3YSZ powders with a particle size of 60–70 nm, pressed into cylindrical compacts with a diameter of 5 mm, a height of 10 mm and a relative density of 50%–55%. All experiments were carried out in ambient air under a constant heating rate of 10 °C/min from room temperature. They placed stainless steel sheets between alumina pushrods and the flat surfaces of the specimens to apply an electrical field. For each experiment, they employed a direct current power source to apply a constant voltage, switching to current-control mode when the current reached a limit of 1 A. They used a camera and a pyrometer to measure the radial and axial deformations and surface temperature of the specimens, respectively.

**Fig. 2:**
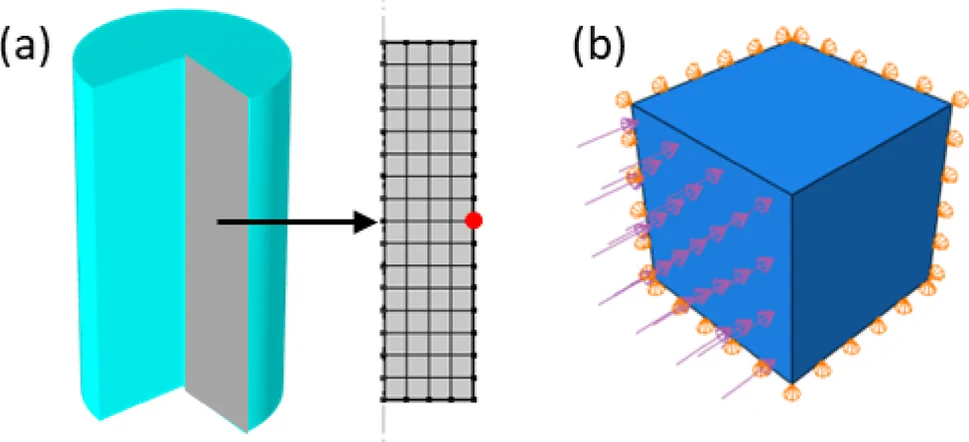
Finite element models for (**a**) cylindrical sample, with position (red point) where surface temperature was evaluated, and (**b**) single element

FE simulations were conducted using the Time-Dependent Solver in COMSOL Multiphysics 6.2 (COMSOL Inc., Sweden) to simulate the electrical, thermal and deformation processes of the experiments. An axisymmetric model for the specimen was constructed to simulate the flash sintering cases (Fig. 2a), whilst a single hexahedral element was used for the conventional sintering case (Fig. 2b). The axisymmetric model was meshed using quadrilateral elements with a size of 0.625 mm. A mesh sensitivity study was carried out and confirmed convergence; therefore, this mesh was used in this study.

### Electrical and thermal analysis

For the flash sintering cases, a terminal boundary condition was applied to the top surface of the specimen to apply a constant voltage during the voltage-control mode, switching to the current-control mode when the current reached 1 A. The bottom surface was connected to the ground. Surface-to-Ambient radiation and surface convection applied to the lateral surface, and surface conduction to the pushrods applied to the top and bottom surfaces. The displacement of the top surface was fixed in the axial direction. Initially, an electrothermal analysis was performed with the measured deformations of the specimens applied to the model for the flash sintering cases.

**Fig. 3:**
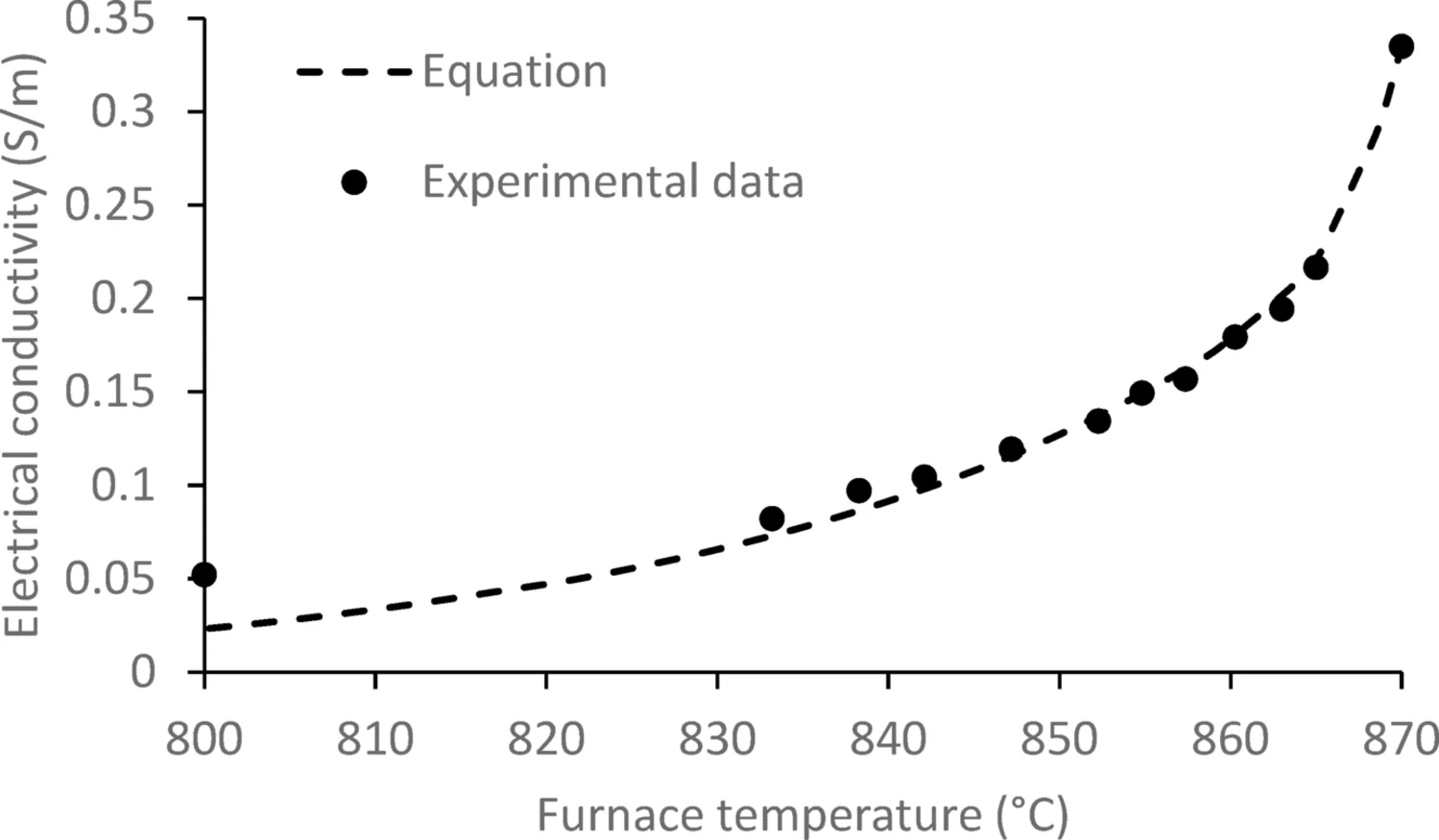
Fitted electrical conductivity vs. furnace temperature plot for 100 V & 5 MPa case, in comparison with experimental data (Francis & Raj, 2012)

In the 100 V & 5 MPa case, measured surface temperature indicated that flash sintering occurred at a furnace temperature of approximately 870 °C. Consequently, the input power data before 870 °C were used to calculate the corresponding electrical conductivity, under the assumption that the sample temperature equalled to the furnace temperature prior to 870 °C. Subsequently, $\:{\varSigma\:}_{g}$, $\:{\varSigma\:}_{f}$, $\:{Q}_{g}$ and $\:{Q}_{f}$ in Eq. (2) were fitted against the calculated electrical conductivity, measured density and temperature using MATLAB R2022a (MathWorks, USA). The fitted parameter values are provided in Table 2. The density of fully dense 3YSZ $\:{\rho\:}_{f}$ was taken as 6086 kg/m^3^ (Schlichting et al., 2001), and the mean value of 0.525 in the range of initial relative density was used as the initial relative density $\:{\rho\:}_{g}^{r}$ in this study. Hence, the density of green body was 3195.15 kg/m^3^. The fitted electrical conductivity is plotted in Fig. 3, in comparison with the corresponding experimental data. It should be noted that, since the furnace temperature increased at a constant rate, it is used to represent the time in some of the figures in this paper.

**Table 2:** Parameter values for electrical conductivity

$\:{\varSigma\:}_{g}$ (S/m)	$\:{\varSigma\:}_{f}$ (S/m)	$\:{Q}_{g}$ (kJ/mol)	$\:{Q}_{f}$ (kJ/mol)	$\:{\rho\:}_{g}$ (kg/m^3^)	$\:{\rho\:}_{f}$ (kg/m^3^)
6.02 × 10^15^	6.01 × 10^15^	362	341	3195.15	6086

**Fig. 4:**
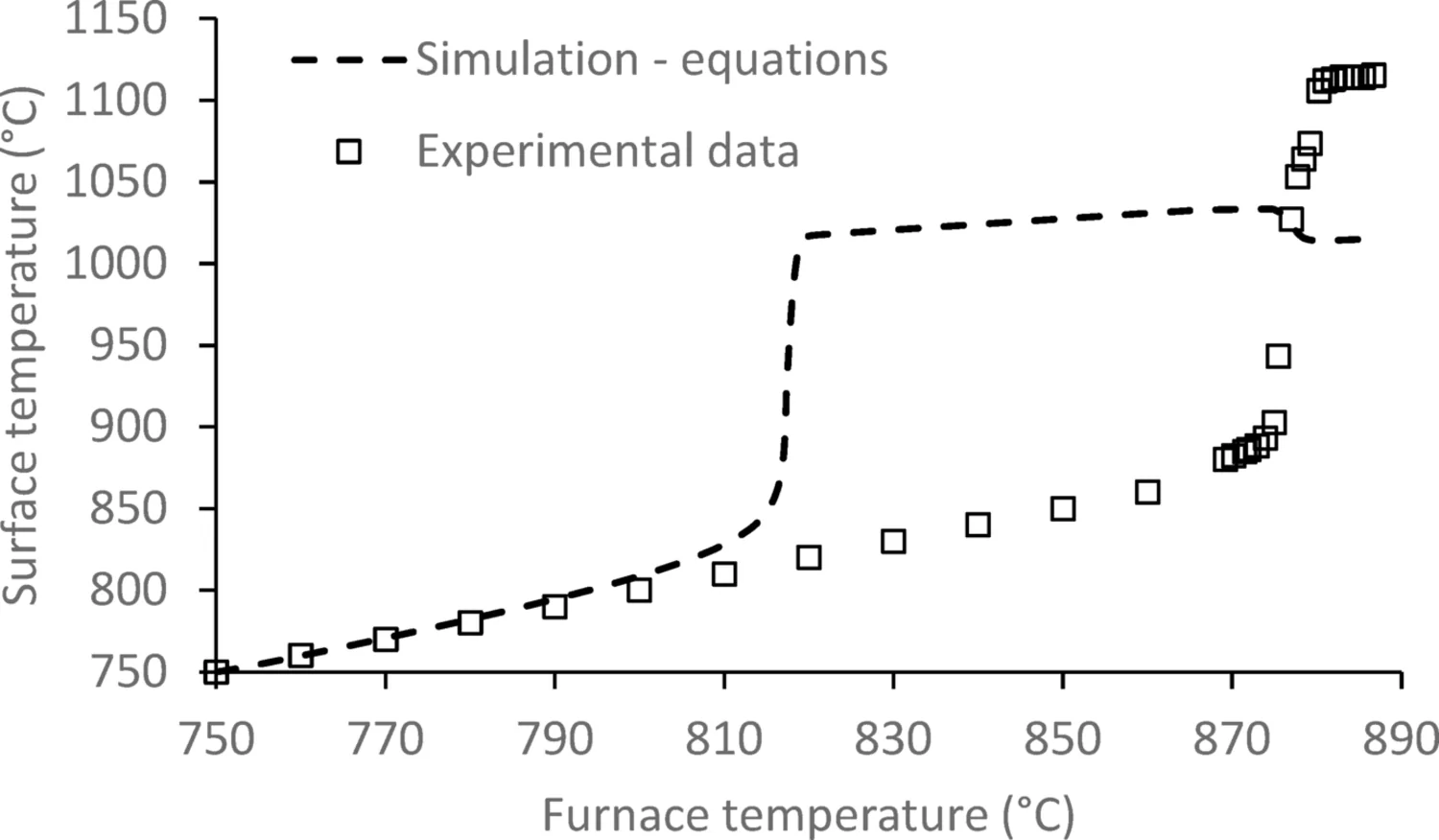
Temporal evolution of surface temperature for 100 V & 5 MPa case, simulated by using Eqs. (1)-(8), in comparison with experimental data (Francis & Raj, 2012)

The values of the specific heat capacity $\:{C}_{p}$ for fully dense 3YSZ and the emissivity *ϵ* were provided in Tanaka et al. (2001), whilst the value of the thermal conductivity for fully dense 3YSZ $\:{k}_{f}$ was obtained from Li et al. (2021). The convective heat transfer coefficient * h* was assumed to be 1.25 W/(m^2^·K) as recommended in ISO 6946:2017. The thermal conductivity for surface conduction to alumina pushrods was taken as 27 W/(m·K) from the built-in material library in COMSOL. Parameter values used in the heat equation are summarised in Table 3. The 100 V & 5 MPa case was simulated using the fitted electrical conductivity and thermal parameters. The surface temperature at the position indicated in Fig. 2a was extracted and plotted against furnace temperature for comparison with experimental data. A significant discrepancy was observed: the FE analysis predicted a much earlier onset of flash sintering (Fig. 4).

**Table 3:** Parameter values for heat equation

$\:{C}_{p}$ (J/(kg K))	$\:{k}_{f}$ (W/(m K))	ε	* h* (W/(m^2^ K))
600	2.5	0.7	1.25

**Fig. 5:**
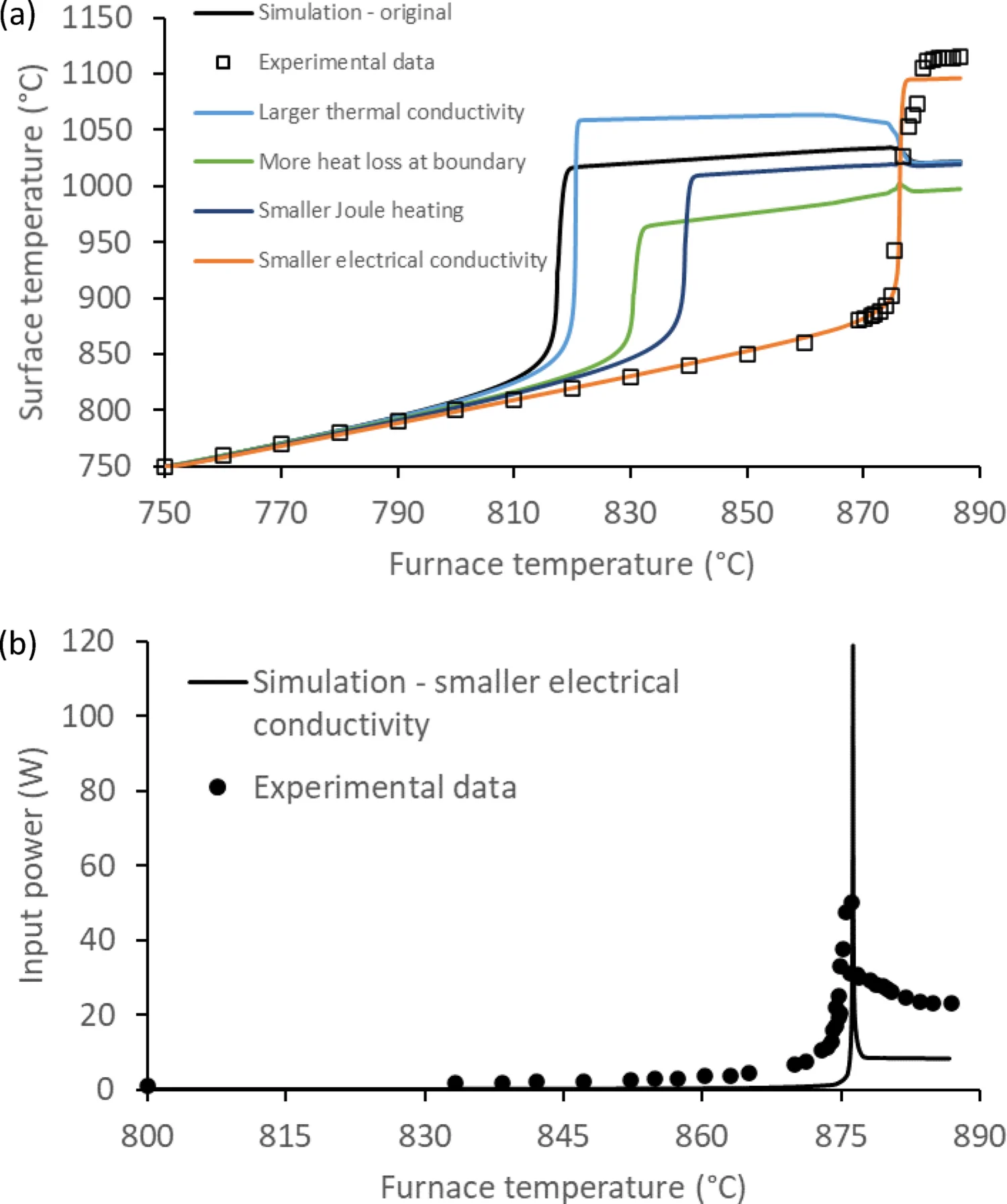
Effects of larger thermal conductivity, more heat loss at boundaries, smaller Joule heating and smaller electrical conductivity on (**a**) delay in flash sintering onset and (**b**) comparison between simulated input power using smaller electrical conductivity and experimental data (Francis & Raj, 2012) for 100 V and 5 MPa case

To delay the sintering onset in the FE analysis for the 100 V & 5 MPa case, multiple parameter adjustments were explored. These included larger thermal conductivity, more heat loss at boundaries, smaller Joule heating and smaller electrical conductivity (Fig. 5a). Among them, the lower electrical conductivity produced the most pronounced effect in delaying the flash sintering onset, suggesting a potential inaccuracy in Eq. (2). However, when comparing the input power calculated using lower electrical conductivity with the experimental data as shown in Fig. 5b, it was observed that the predicted input power was considerably lower than the measured data. These findings indicated that adjusting the electrical conductivity alone was insufficient to match the FE results with the experimental data. The assumption made in the expression for Joule heating, Eq. (8), was also questionable.

### Deformation analysis

**Fig. 6:**
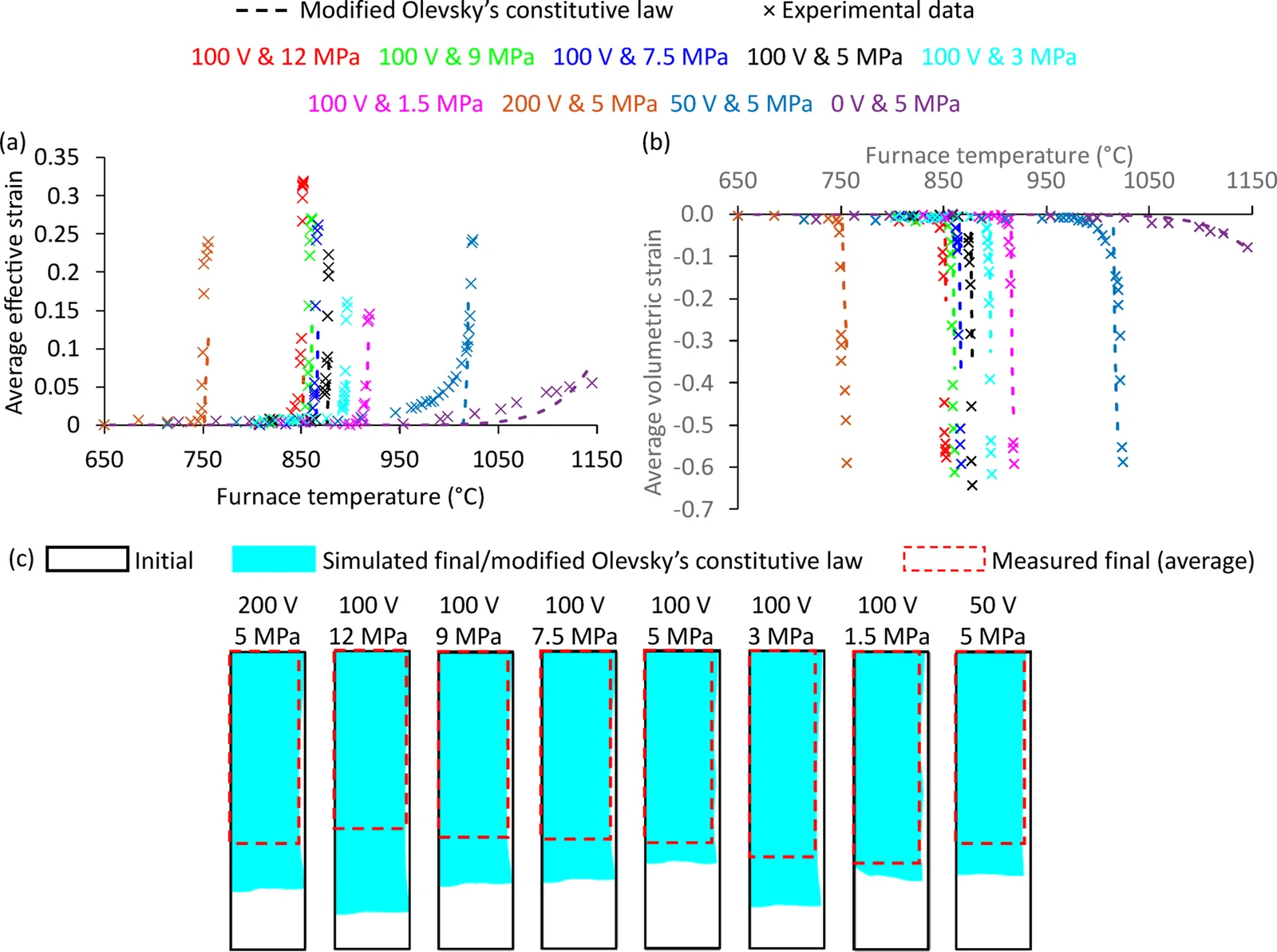
Temporal evolutions of (**a**) effective strains and (**b**) volumetric strains averaged over sample volume (dashed lines: finite element predictions using COMSOL with modified Olevsky’s constitutive law; discrete symbols: experimental data provided in Francis and Raj (2012); (**c**) comparison between initial shapes, simulated final shapes using modified Olevsky’s constitutive law and measured final shapes (average) for flash sintering cases

For the deformation analysis, sample temperature and temperature rate were required as inputs. Instead of using the inaccurate FE results presented in the Electrical and thermal analysis, correct results obtained using the right electrical conductivity and heat source (presented later in the Finite element analysis with nested ANNs replacing electrical conductivity and Joule heating) were used. A pressure was applied to the bottom surface of the specimen. For the model parameters of the modified Olevsky’s constitutive law, the activation energy for densification $\:{C}_{s2}$ for 3YSZ was provided in Mazaheri et al. (2009). Remaining parameters were determined by our best effort in fitting the simulated average effective and volumetric strains to the experimental data for all cases. The parameter values for the modified Olevsky’s constitutive law are provided in Table 4, with the corresponding simulation results compared to the experimental data in Fig. 6. Very large discrepancies could be observed between the predicted and measured final shapes at the end of flash sintering process as in Fig. 6c. It should be noted that, since only two strain measurements were available in the radial and longitudinal directions at each time point in Francis and Raj (2012), the values had to be interpreted as average strains. Consequently, the “measured” final shapes shown in Fig. 6c represent average final shapes rather than true final shapes.

**Table 4:** Parameter values for modified Olevsky’s constitutive law of flash sintering

$\:{C}_{a}$ (Pa s)	$\:{C}_{b}$ (K/s)	$\:{C}_{c}$ (Pa s)	*n*	$\:{C}_{s2}$ (kJ/mol)	* d* (nm)	$\:\gamma\:$ (J/m^2^)	ξ	N_s_
6.67 × 10^7^	1.2	3.44 × 10^7^	2	485	65	1	307.69	1

Some possible inaccuracies may exist in the measurements of surface temperature and sintering deformation with the pyrometer and optical techniques used by Francis and Raj (2012). However, the discrepancies between the FE predictions and the measured data were so large that improved measuring accuracy would not alter the fact that theories presented in the Mathematical equations for flash sintering required major improvement.

## Nested machine learning

In the present study, it was proposed that the empirical rules - specifically electrical conductivity (Eq. (2)), Joule heating (Eq. (8)) and the constitutive law - were each replaced by an ANN. The architectures of the ANNs are provided in Table 5.

**Table 5:** Architectures of ANNs

	Input variables	Output variables	Number of layers	Number of neurons in each hidden layer	Activation function
Electrical conductivity (Eq. (2))	Temperature Relative density	Electrical conductivity	5	1000	Rectified linear unit (ReLU) for hidden layers, linear for output layer
Joule heating (Eq. (8))	Magnitude of current density Magnitude of electric field strength	Joule heating			
Constitutive law	Relative density Von Mises stress Mean stress Temperature Temperature rate	Effective strain rate Volumetric strain rate			

These ANNs were firstly trained in standalone mode using data generated from Eqs. (2), (8) and the modified constitutive law. They were subsequently retrained while nested inside COMSOL, using experimental data of input power, surface temperature and strains. In COMSOL, analytical expressions such as Eqs. (2), (8) and the constitutive law can be defined via user-implemented MATLAB functions. The forward propagation of an ANN is simply a complex analytical expression involving scalers, weights, biases and activation functions. Therefore, an ANN can be implemented as a MATLAB function in COMSOL. This approach was taken in our work and referred to as nesting an ANN in the FE analysis. A new backpropagation algorithm is presented to enable the second-step training of ANNs nested in the FE.

### Backpropagation algorithm for training ANNs nested in a finite element package

As an example, the nested training of an ANN that replaces the constitutive law is demonstrated this section. For the forward propagation, the training dataset for the ANN is denoted as $\:\left\{\left({\boldsymbol{x}}^{1},{\boldsymbol{y}}^{1}\right),\dots\:,\left({\boldsymbol{x}}^{c},{\boldsymbol{y}}^{c}\right)\right\}$, where $\:\boldsymbol{x}$ and $\:\boldsymbol{y}$ represent the vectors for the scaled input and output variables, i.e., $\:\boldsymbol{x}$ = scaled [relative density, von Mises stress, mean stress, temperature, temperature rate] and $\:\boldsymbol{y}$ **=** scaled [effective strain rate, volumetric strain rate], respectively. For each $\:\left({\boldsymbol{x}}^{k},{\boldsymbol{y}}^{k}\right)$ ($\:1\le\:k\le\:c)$, the components of the vectors in the hidden and output layers are calculated by

10 $$ z_{j}^{{lk}} = f\left( {z0_{j}^{{lk}} } \right) = f\left( {\mathop \sum \limits_{i} \left( {w_{{ji}}^{l} z_{i}^{{\left( {l - 1} \right)k}} } \right) + b_{j}^{l} } \right). $$

where * l* indicates the layer, $\:f\left(z\right)$ is the activation function and $\:{w}_{ji}^{l}$ and $\:{b}_{j}^{l}$ are the components of the weights and biases in the * l* layer, respectively. Hence, $\:{z}_{i}^{1k}={x}_{i}^{k}$ and $\:{z}_{i}^{ok}={\widehat{y}}_{i}^{k}$, where $\:{\widehat{y}}_{i}^{k}$ is the ANN’s estimated $\:{y}_{i}^{k}.$

The ANN replacing the constitutive law that nested in the FE analysis is trained by minimising the mean squared error

11 $$ \begin{gathered} J\left( {\left( {{{{\hat{\varepsilon}}_{{{{{e}}_{{scale}}}}} }} - {\varepsilon }_{{{{e}_{scale}}}} } \right),\left( {{{{\hat{\varepsilon }}_{{{{v}_{scale}}}} }} - {\varepsilon }_{{{{v}_{scale}}}} } \right)} \right) = \hfill \\ \frac{1}{{4N}}\mathop \sum \limits_{{a = 1}}^{N} \left[ {\left( {{{\hat{\varepsilon}} }_{{{e}_{scale}}}^{a} - {\varepsilon} _{{{e}_{scale}}}^{a} } \right)^{2} + \left( {{{{\hat{\varepsilon}}  }}_{{{\nu}_{scale}}}^{a} - {\varepsilon} _{{{\nu}_{scale}}}^{a} } \right)^{2} } \right] \hfill \\ \end{gathered} .$$

where * N* is the total number of experimental data and $\:{{{\varepsilon}}_{{\boldsymbol{e}_{scale}}}}$ and $\:{{{\varepsilon}}_{{\boldsymbol{v}_{scale}}}}$ are the effective and volumetric strains scaled by min-max scaling, respectively. Specifically, $\:{{{{\hat{\varepsilon}}}_{{\boldsymbol{e}_{scale}}}}}$ and $\:{{{{\hat{\varepsilon}}}_{{\boldsymbol{v}_{scale}}}}}$ are obtained from the FE analysis with the nested ANN.

Each iteration of gradient descent updates the weights according to the Adam algorithm (Zhang, 2019) as

12 $$ w_{{ijnew}}^{l}  = w_{{ijold}}^{l}  - \alpha \left( {\frac{{\tilde{m}^{l} _{{ijnew}} }}{{\sqrt {\tilde{v}_{{ijnew}}^{l} } }} + e} \right),  $$

where $\:\alpha\:$ is the learning rate and * e* is a very small number to prevent any division by zero in the implementation. We also have

13 $$  \tilde{m}_{{ijnew}}^{l}  = \frac{{m_{{ijnew}}^{l} }}{{1 - \beta _{1} }},  $$

14 $$  \tilde{v}_{{ijnew}}^{l}  = \frac{{v_{{ijnew}}^{l} }}{{1 - \beta _{2} }},$$

with

15 $$\:\begin{array}{c}{{m}_{ij}^{l}}_{new}={\beta\:}_{1}{{m}_{ij}^{l}}_{old}+\left(1-{\beta\:}_{1}\right){{J}^{{\prime\:}}}_{ij}^{l},\end{array}$$

16 $$\:\begin{array}{c}{{v}_{ij}^{l}}_{new}={\beta\:}_{2}{{v}_{ij}^{l}}_{old}+\left(1-{\beta\:}_{2}\right){\left({{J}^{{\prime\:}}}_{ij}^{l}\right)}^{2},\end{array}$$

where $\:{\beta\:}_{1}$ and $\:{\beta\:}_{2}$ are the exponential decay rates for the first-moment and second-moment estimates, respectively, and $\:{{J}^{{\prime\:}}}_{ij}^{l}$ is the partial derivative of * J* with respect to the weights in the * l* layer. Initially, both $\:{m}_{ij}^{l}$ and $\:{v}_{ij}^{l}$ are set to 0. $\:{{J}^{{\prime\:}}}_{ij}^{l}$ can be written as (Hecht-Nielsen, 1989)

17 $$\:\begin{array}{c}{{J}^{{\prime\:}}}_{ij}^{l}=\sum\:_{k=1}^{c}{\delta\:}_{i}^{l}\frac{\partial\:{z}_{i}^{lk}}{\partial\:{w}_{ij}^{l}},\end{array}.$$

with

18 $$\:\begin{array}{c}{\delta\:}_{i}^{l}=\sum\:_{b=1}^{{r}^{l+1}}{\delta\:}_{b}^{\left(l+1\right)}\frac{\partial\:{z}_{b}^{\left(l+1\right)k}}{\partial\:{z}_{i}^{lk}},\end{array}.$$

where $\:{r}^{l}$ is the number of vector components in the * l* layer. Considering the output layer, we have

19 $$ \left\{ {\begin{array}{*{20}c}    \begin{gathered}   \delta _{1}^{o}  = \mathop \sum \limits_{{k = 1}}^{c} \frac{{\partial J}}{{\partial \hat{y}_{1}^{k} }} \hfill \\    = \mathop \sum \limits_{{a = 1}}^{N} \left\{ {\frac{{\partial J}}{{\partial \left( {\widehat{\varepsilon }_{{escale}}^{a}  - \varepsilon _{{escale}}^{a} } \right)}}\mathop \sum \limits_{{k = 1}}^{c} \frac{{\partial \widehat{\varepsilon }_{{escale}}^{a} }}{{\partial \hat{y}_{1}^{k} }}} \right\}, \hfill \\  \end{gathered}   \\    \begin{gathered}   \delta _{2}^{o}  = \mathop \sum \limits_{{k = 1}}^{c} \frac{{\partial J}}{{\partial \hat{y}_{2}^{k} }} \hfill \\    = \mathop \sum \limits_{{a = 1}}^{N} \left\{ {\frac{{\partial J}}{{\partial \left( {\widehat{\varepsilon }_{{\nu scale}}^{a}  - \varepsilon _{{\nu scale}}^{a} } \right)}}\mathop \sum \limits_{{k = 1}}^{c} \frac{{\partial \widehat{\varepsilon }_{{\nu scale}}^{a} }}{{\partial \hat{y}_{2}^{k} }}} \right\}. \hfill \\  \end{gathered}   \\   \end{array} } \right. $$

Calculating $\:\partial\:{{{\hat{\varepsilon}}^{a}_{{e}_{scale}}}}/\partial\:{\widehat{y}}_{1}^{k}$ and $\:\partial\:{{{\hat{\varepsilon}}^{a}_{{v}_{scale}}}}/\partial\:{\widehat{y}}_{2}^{k}$ in Eq. (19) is effectively to differentiate the scaled strains, ($\:{{{\hat{\varepsilon}}^{a}_{{e}_{scale}}}},{{{\hat{\varepsilon}}^{a}_{{v}_{scale}}}}$), which are obtained in the FE analysis with respect to the outputs of the ANN, ($\:{\widehat{y}}_{1}^{k}$, $\:{\widehat{y}}_{2}^{k}$), that replaced the constitutive law. These calculations are difficult because the relationships between them are complex and numerical. However, in a separate work (see Acknowledgement), we realised that $\:{\delta\:}_{i}^{l}$ would always multiply the learning rate $\:\alpha\:$ in the backpropagation. The learning rate $\:\alpha\:$ is an empirically adjusted value in the machine learning algorithm. Consequently, the precise values of $\:\partial\:{{{\hat{\varepsilon}}^{a}_{{e}_{scale}}}}/\partial\:{\widehat{y}}_{1}^{k}$ and $\:\partial\:{{{\hat{\varepsilon}}^{a}_{{v}_{scale}}}}/\partial\:{\widehat{y}}_{2}^{k}$ become irrelevant, whilst their signs play a critical role. It is therefore possible to ignore the absolute values of $\:\partial\:{{{\hat{\varepsilon}}^{a}_{{e}_{scale}}}}/\partial\:{\widehat{y}}_{1}^{k}$ and $\:\partial\:{{{\hat{\varepsilon}}^{a}_{{v}_{scale}}}}/\partial\:{\widehat{y}}_{2}^{k}$, and rewrite Eq. (19) into

20 $$\:\begin{array}{c}\left\{\begin{array}{c}{\delta\:}_{1}^{o}=\pm\:\sum\:_{a=1}^{N}\frac{\partial\:J}{\partial\:\left({{{\hat{\varepsilon}}^{a}_{{e}_{scale}}}}-{{{\varepsilon}^{a}_{{e}_{scale}}}}\right)},\\\:{\delta\:}_{2}^{o}=\pm\:\sum\:_{a=1}^{N}\frac{\partial\:J}{\partial\:\left({{{\hat{\varepsilon}}^{a}_{{v}_{scale}}}}-{{{\varepsilon}^{a}_{{v}_{scale}}}}\right)}.\end{array}\right.\end{array}.$$

    

where the sign of $\:\pm\:$ is determined based on our understanding for relationships between $\:({{{\hat{\varepsilon}}^{a}_{{e}_{scale}}}},\:{{{\hat{\varepsilon}}^{a}_{{v}_{scale}}}})$ and ($\:{\widehat{y}}_{1}^{k}$, $\:{\widehat{y}}_{2}^{k}$). In the case of sintering deformation, it is apparent that a faster strain rate of the constitutive law leads to a larger sintering strain in the FE analysis, that is, the signs in Eq. (20) have to be positive.

The above algorithm and argument are in fact generic and apply to other problems. For the ANN replacing electrical conductivity, considering Eq. (1) and the relation of input power = voltage × current, an increase in electrical conductivity results in an increase in input power under the voltage-control mode and a decrease under the current-control mode. Consequently, the sign in Eq. (20) was determined as + in the voltage-control mode and – in the current-control mode. For the ANN replacing Joule heating, an increase in Joule heating results in an increase in surface temperature, and, thus, the sign was determined as +. Similar to the weights, the biases can be updated by

21 $$  b_{{inew}}^{l}  = b_{{iold}}^{l}  - \alpha \left( {\frac{{\tilde{m}_{{inew}}^{l} }}{{\sqrt {\tilde{v}_{{inew}}^{l} } }} + e} \right). $$

Table 6 provides a step-by-step summary of the backpropagation algorithm.

**Table 6:**
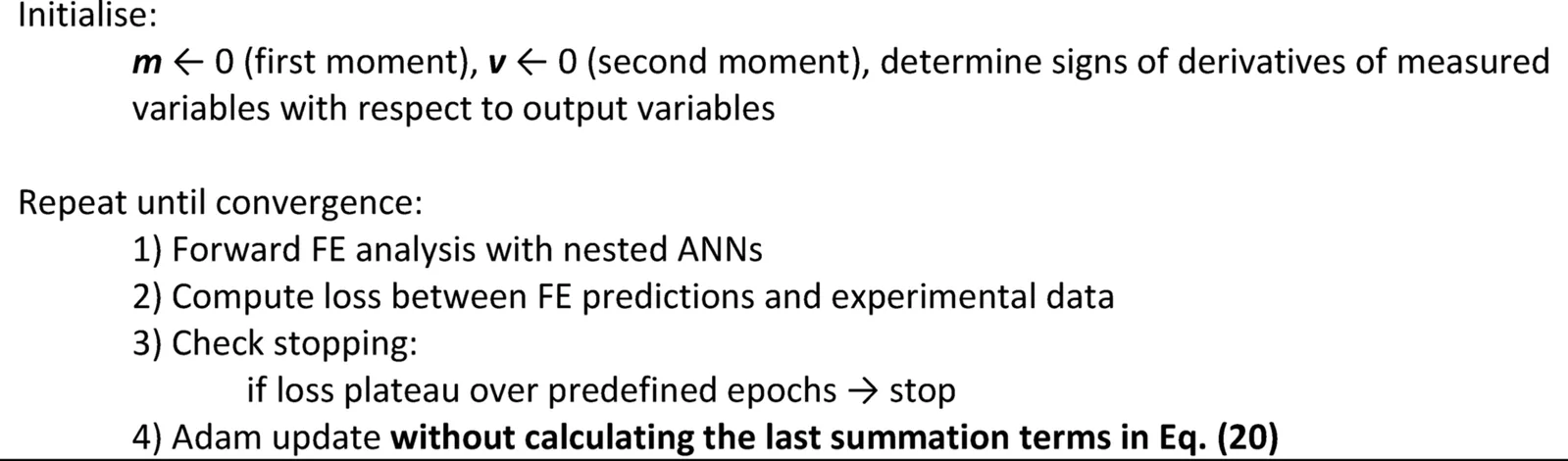
Summary of proposed backpropagation algorithm

### Two-step training of ANNs

During the pretraining (first-step training), all ANNs were developed using the Keras-TensorFlow framework. For the ANNs replacing electrical conductivity and Joule heating, data scaling for the input and output variables was achieved using the MinMax Scaler and Quantile Transformer from Python’s sklearn library, respectively. The ANN replacing constitutive law employed the Quantile Transformer for both input and output scaling. For the Quantile Transformer, the sizes of both subsample and n_quantiles were chosen to be the same as the size of the training datasets. Data were split into training, validation and test sets in an 80:10:10 ratio using the test/train split function in the scikit library. The Adam optimiser was employed (*β*_*1*_ = 0.9, *β*_*2*_ = 0.999, *e* = 10^− 8^), with the mean squared error of the output variables as the loss function. The fix seed was set to 1 for all ANNs. Trainings were conducted with a batch size of 256 and an early stopping criterion of 1000 epochs. The ANN replacing electrical conductivity was trained for 3000 epochs with a learning rate of 0.001. Whilst the learning rates for the ANNs replacing Joule heating and constitutive law were initially set to 0.001 and reduced to 0.0001 upon triggering an early stopping criterion of 1000 epochs. The epochs after early stopping for learning rates of 0.01 and 0.001 were 8111 and 7344 for ANN replacing Joule heating and 2975 and 3053 for ANN replacing constitutive law, respectively.

Pretraining datasets were generated from Eqs. (2), (8) and the modified constitutive law using MATLAB for the ANNs replacing electrical conductivity, Joule heating and constitutive law, respectively. A total of 100,000 input variable datasets were randomly generated in MATLAB for each ANN. For retraining with experimental data, a master MATLAB script was developed to execute the nested training process. This script performed FE simulations using the nested ANNs, extracted simulation results, calculated the mean squared error between the simulation results and the experimental data, and updated the weights and biases of the ANNs via the backpropagation algorithm described in the Backpropagation algorithm for training ANNs nested in a finite element package. Experimental data of input power were used to retrain the ANN replacing electrical conductivity (cases: 100 V & 12, 9, 7.5, 5, 3, 1.5 MPa), whilst surface temperature data (100 V & 5 MPa) were used for the ANN replacing Joule heating. Effective and volumetric strains from all cases were used to retrain the ANNs replacing constitutive law. It should be noted that data of input power and surface temperature for cases not mentioned above were unavailable in Francis and Raj (2012). The same scalers used during pretraining were applied in the retraining process. Each epoch took around 4 h with a processor of 11th Gen Intel(R) Core(TM) i7-1185G7 @ 3.00 GHz.

## Results of finite element analysis with nested ANNs

### Finite element analysis with nested ANNs replacing electrical conductivity and joule heating

**Fig. 7:**
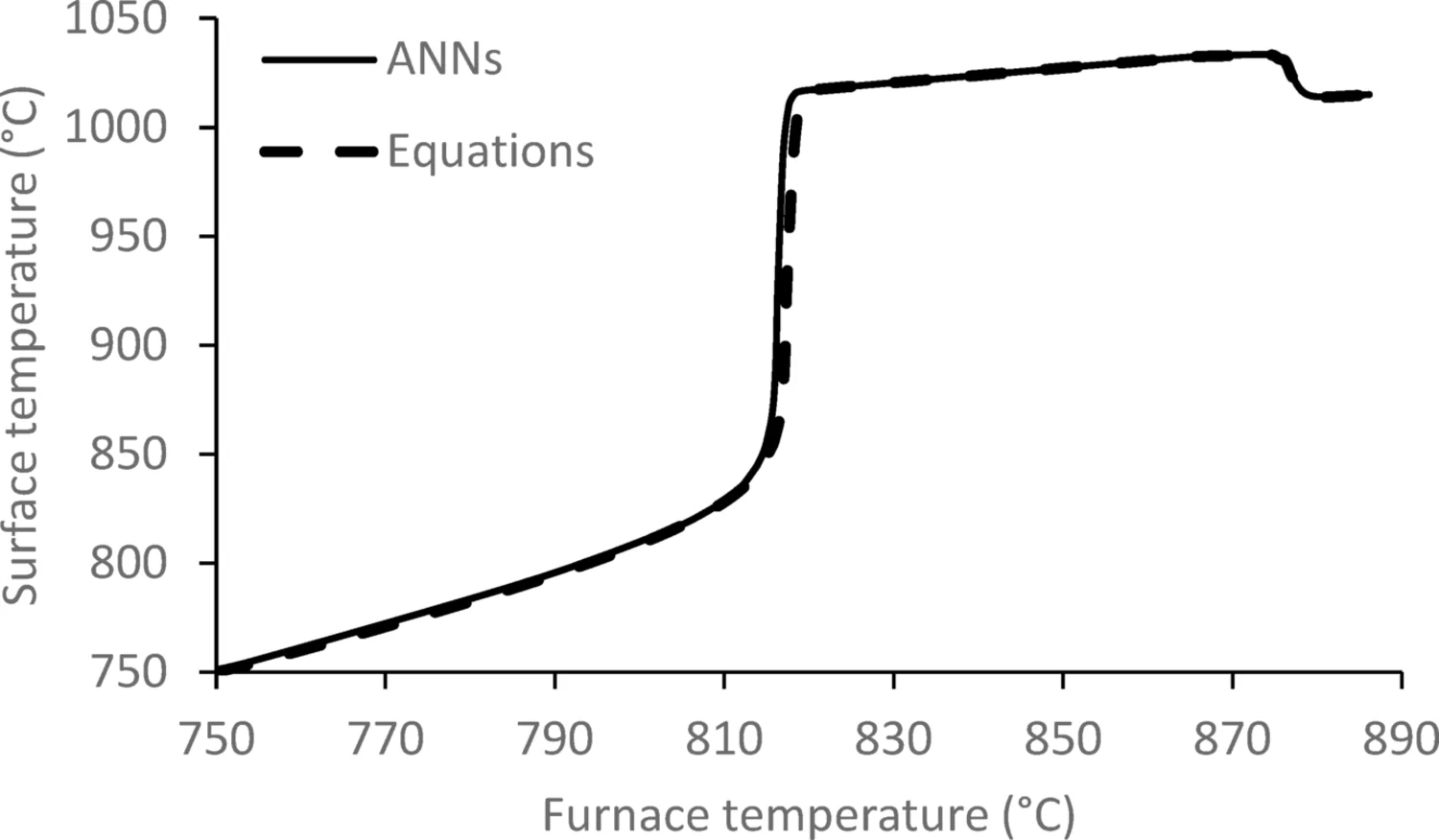
Temporal evolutions of surface temperature for 100 V & 5 MPa case simulated using (i) pretrained ANNs replacing electrical conductivity and Joule heating and (ii) Eqs. (2) and (8)

The performance of the pretrained ANNs replacing electrical conductivity and Joule heating was first evaluated. Figure 7 compares the temporal evolutions of surface temperature for the 100 V & 5 MPa case, simulated using (i) the pretrained ANNs and (ii) Eqs. (2), (8). The close agreement between the two indicated that the pretrained ANNs successfully reproduced the behaviours described by the original equations.

**Fig. 8:**
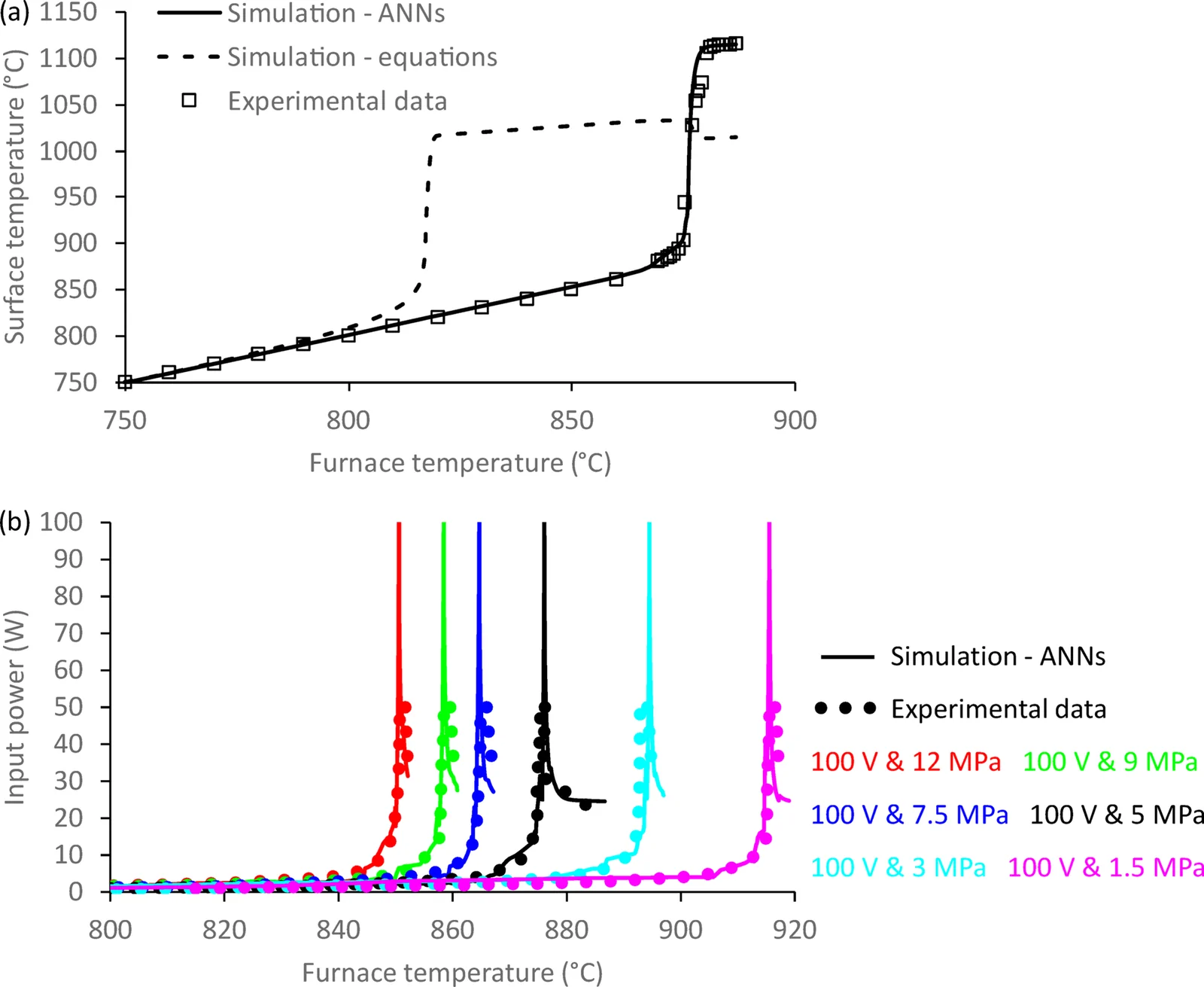
Temporal evolutions of (**a**) surface temperature for 100 V & 5 MPa case and (**b**) input power for 100 V cases, simulated by retrained ANNs replacing electrical conductivity and Joule heating, in comparison with experimental data (Francis & Raj, 2012)

To examine the effect of retraining, Fig. 8a shows the simulate surface temperature evolution for the same case (100 V & 5 MPa) obtained with the retrained ANNs. Retraining substantially delayed the predicted onset of flash sintering, yielding results in close agreement with experiment. The temporal evolutions of input power predicted for 100 V cases are shown in Fig. 8b. The retrained ANNs captured the experimental data well, thereby validating both the nested training strategy and the feasibility of the proposed framework to multiphysics problems.

It should be noted that Francis and Raj (2012) reported input power data only up to 50 W. However, given the initial applied voltage of 100 V and the current limit of 1 A, the peak input power would be expected to reach at least 100 W, consistent with the simulation results.

**Fig. 9:**
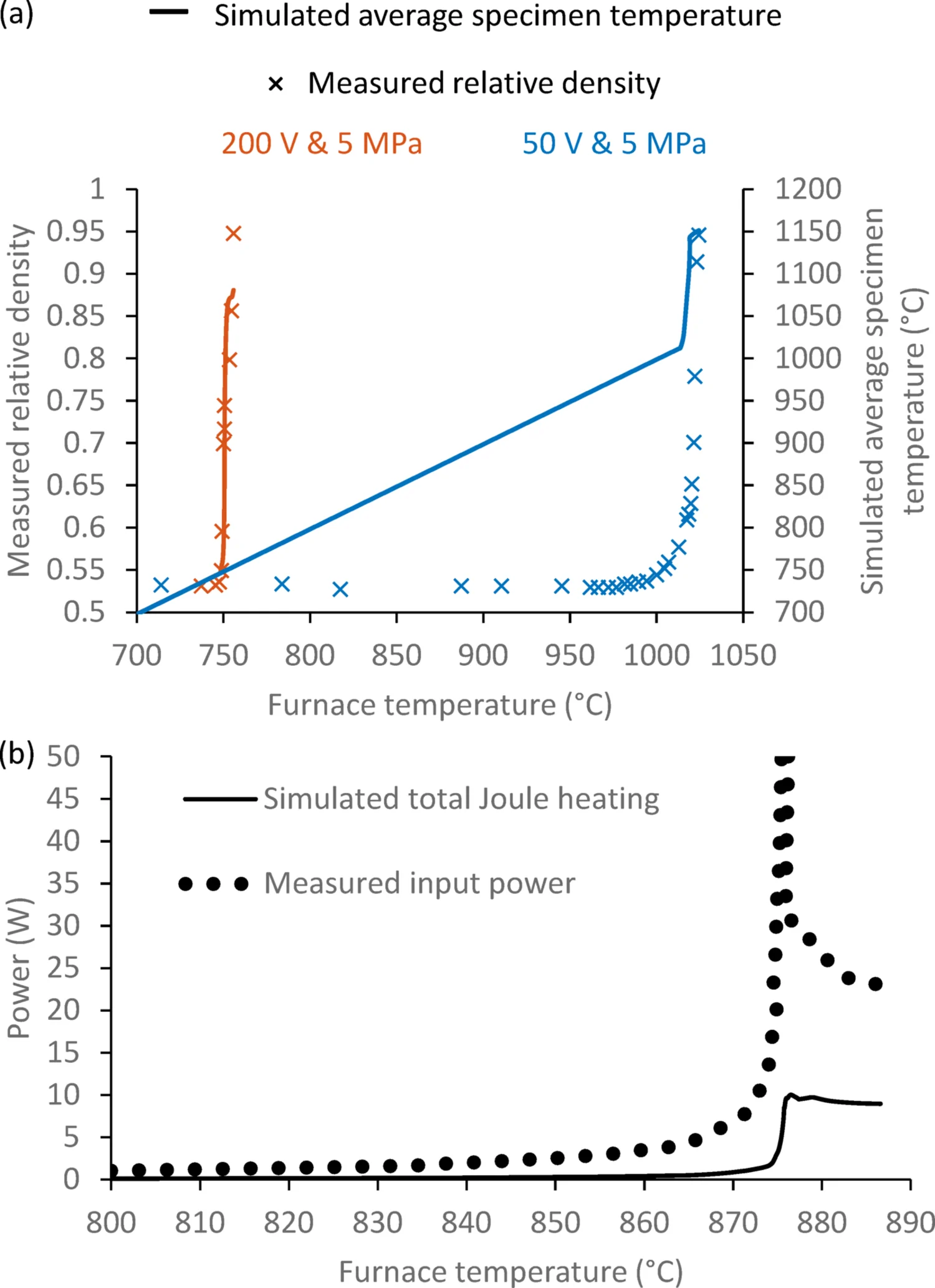
(**a**) Temporal evolutions of average sample temperature for 200 V & 5 MPa and 50 V & 5 MPa cases, simulated by retrained ANNs for electrical conductivity and Joule heating, in comparison with measured relative density (Francis & Raj, 2012); (**b**) temporal evolution of total Joule heating for 100 V and 5 MPa case, simulated by retrained ANNs for electrical conductivity and Joule heating, in comparison with measured input power (Francis & Raj, 2012)

The retrained ANNs were also applied to the 200 V & 5 MPa and 50 V & 5 MPa cases. Because only deformation data were experimentally available for these two cases (Francis & Raj, 2012), the simulated average specimen temperatures were compared with the measured relative densities (Fig. 9a). The surges in the simulated average temperature occurred at the same furnace temperatures as those in the measured relative densities, indicating that the predicted onsets of flash sintering aligned well with the measurements. Furthermore, for the 100 V and 5 MPa case, the simulated total Joule heating in the specimen was extracted and compared to the measured input power (Fig. 9b). The total Joule heating was markedly lower than the input power, implying that a substantial portion of the supplied electrical energy was dissipated into the furnace rather than being converted to internal thermal energy.

### Finite element analysis with nested ANN replacing constitutive law

**Fig. 10:**
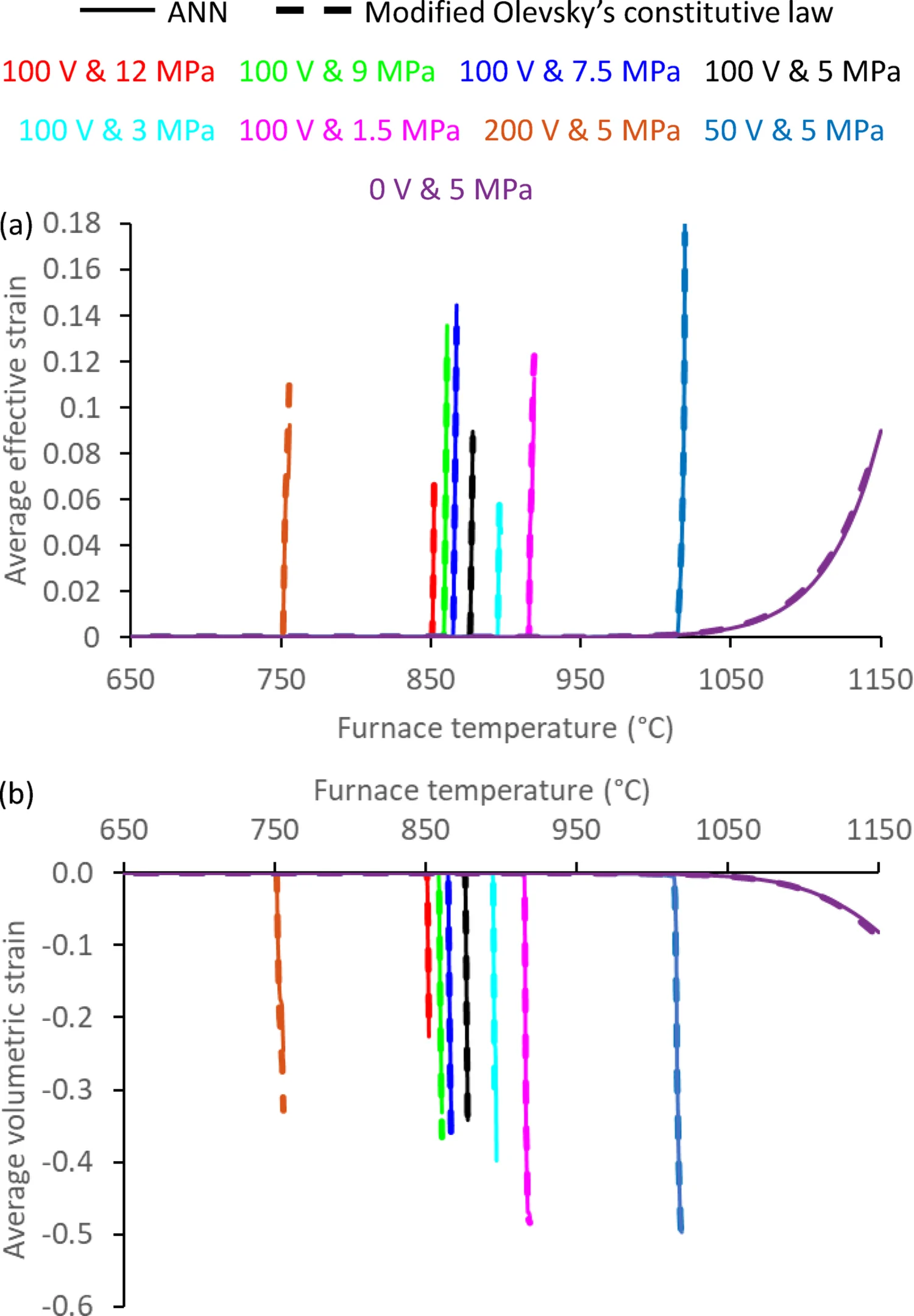
Temporal evolutions of (**a**) effective strain and (**b**) volumetric strain, both averaged over the volume of the sample for all cases, simulated with pre-trained ANN replacing constitutive law (solid lines) and with modified Olevsky’s constitutive law (dashed lines)

Figure 10 compares the predictions for average effective and volumetric strains obtained from FE analysis using the pretrained ANN replacing constitutive law with those predicted using the modified Olevsky’s constitutive law. The close agreement between the two sets of results validated the pretraining process of the ANN replacing constitutive law.

**Fig. 11:**
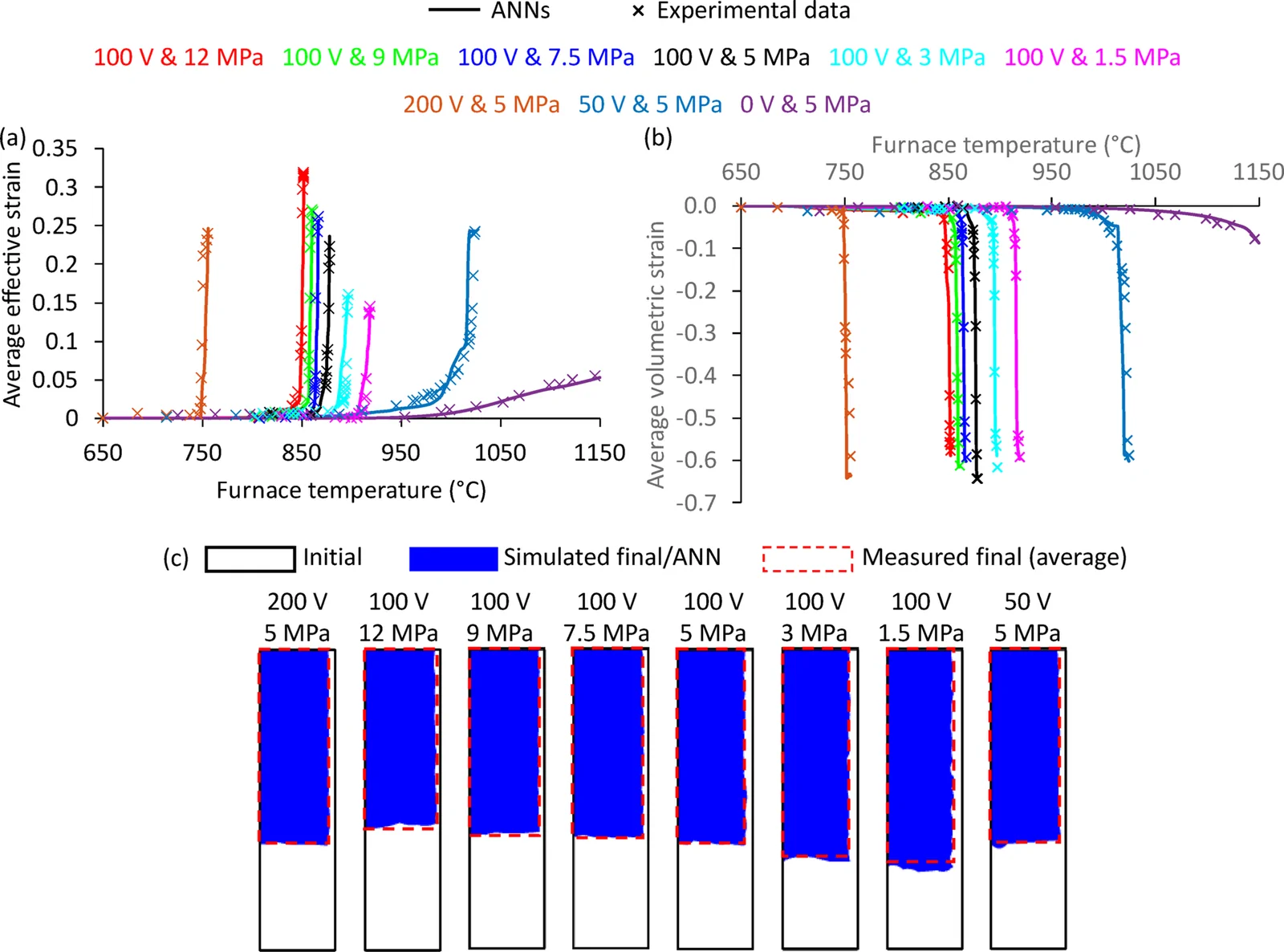
Temporal evolutions of average (**a**) effective and (**b**) volumetric strains for all cases, simulated by retrained ANNs, in comparison with experimental data (Francis & Raj, 2012); (**c**) comparison between initial shapes, simulated final shapes using retrained ANNs and measured final shapes (average) for flash sintering cases

After retrained with experimental strain data, the ANN provided predictions that closely matched the measured temporal evolutions of both effective and volumetric strains across all applied stresses (Fig. 11a and b, respectively). The mean squared error between the predicted and measured strains decreased from 0.0316 (modified Olevsky’s constitutive law) to 0.0034 (retrained ANNs). Figure 11c further compares the predicted and average measured final specimen shapes at the end of flash sintering. The results indicated that the ANNs nested in the FE analysis led to substantial improvements in prediction accuracy, especially in the predicted final shapes. The systematic agreement with experimental strain evolutions and final shapes further validated the feasibility of nested training and improved prediction accuracy of the proposed framework relative to conventional models.

## Discussion

In this study, a multiphysics FE framework for flash sintering was developed by replacing the empirical expressions for electrical conductivity, Joule heating and sintering behaviour with ANNs. The feasibility of training ANNs while nested within a commercial FE package was demonstrated, establishing a pathway toward adaptive digital twin. A critical aspect of this study lay in recognising that the conventional empirical models available in the literature, though widely used, were not sufficiently accurate. The central contribution of this work was to demonstrate that accuracy could be significantly improved by replacing them with ANNs in the FE framework. Validation of the ANN-nested FE approach was carried out against the experimental measurements reported by Francis and Raj (2012). The retrained ANNs produced predictions of sintering strain evolutions (Fig. 11) that closely matched the experimental data, with the mean squared error reduced by an order of magnitude compared to conventional constitutive laws. In addition, essential physical features—including the onset of flash sintering and the final shapes—were consistently captured. These systematic comparisons confirmed that integration of machine learning enabled accurate and reliable predictive capability.

Compared with earlier flash sintering models, the proposed approach captured multiphysics interactions more comprehensively. Most prior studies (Grasso et al., 2011; Mazo et al., 2023; Ni et al., 2022; Pereira Da Silva et al., 2015; Serrazina et al., 2019, 2022; Serrazina, Senos, Serrazina et al., 2020a, b; Serrazina, Vilarinho, Serrazina et al., 2020a, b; Xiao et al., 2023; Yoshida et al., 2018) primarily focused on temperature distributions and neglected deformation. Li et al. (2021) and Arya et al. (2022) incorporated densification, but their assumptions of isotropic shrinkage or master sintering behaviour restricted accuracy. Only two studies attempted to capture deformation: Wang et al. (2023) reported significant discrepancies between simulated and measured densities, whilst Koutoati et al. (2024) presented no experimental validation. By contrast, the present ANN-nested framework learned from measured input power, surface temperature and strain, yielding predictions with an order-of-magnitude lower error than conventional constitutive laws. This demonstrated the value of ANNs in narrowing the persistent gap between FE modelling and experimental observations.

The findings also highlighted the important role of electrical contact resistance. Comparison of simulated Joule heating with measured input power revealed that a large fraction of energy was dissipated at the electrode-sample interfaces, consistent with independent experimental evidence (Ingraci Neto et al., 2021; Jones et al., 2021). The resistance, caused by imperfect contact surfaces, restricts current flow and results in localised heat generation that does not contribute to bulk sintering. Consequently, a large portion of the input energy is dissipated as at the contact surfaces, rather than being effectively utilised for sintering. This study showed that in the experiment carried out by Francis and Raj (2012), approximately 85% of the input energy was dissipated during the voltage-control mode, which decreased to about 60% in the current-control mode. Importantly, the electrical contact resistance was not considered in the electrothermal analysis using Eqs. (2) and (8), which led to discrepancies between simulated and experimental results. The retrained ANN replacing Joule heating captured the energy dissipation effect due to electrical contact resistance, accurately replicating the surface temperature obtained in the experiment.

Another key outcome concerned the effect of heating rate. Although various hypotheses were proposed to explain rapid densification during flash sintering, the definitive mechanism remains uncertain (Ren et al., 2020). Early theories attributed rapid densification to the high temperature generated by Joule heating. However, Raj (2012) demonstrated that the sample temperature induced by Joule heating under an electric field was insufficient to achieve the rapid sintering rate observed. The present findings aligned with this conclusion – the highest temperature achieved in simulations using retrained ANNs was 1169 °C. Since the relative density in the conventional sintering case (0 V & 5 MPa) was only 0.568 at 1145 °C, the temperature during flash sintering was insufficient for achieving full densification in a matter of seconds. This led to an alternative hypothesis - rapid temperature rate. Prior studies by Zhang et al. (2017) and Ji et al. (2017) concluded that rapid temperature rate played a crucial role in the rapid densification, a finding also confirmed in this study. Rapid heating is known to suppress microstructural coarsening caused by surface diffusion, such as grain growth (Francis & Raj, 2012) and neck growth, which otherwise compete with densification (Johnson, 1989). This study further demonstrated the feasibility of incorporating the temperature rate into the constitutive relationship to simulate flash sintering. The modified Olevsky’s constitutive law proposed in this study offers an option for researchers who still want to use a constitutive law rather than an ANN to simulate the flash sintering.

The present results also provide insight into the expected domain of validity of the ANN models. It is often argued that an ANN is only valid within the range of its training data. Whilst this limitation exists, the validity of the ANNs used in this study is at least as good as the analytical equations they replaced. First, the pretraining datasets were generated using these analytical expressions across the full range of expected operating conditions during the real sintering process. Second, the original equations themselves have been validated against only limited and individual experimental data, such as those provided in Francis and Raj (2012). Third, the nested ANNs can be further retrained as new data become available, avoiding the need to start from scratch, making it ideal for a digital twin. This can be contrasted to the conventional mathematical models which have to be recalibrated or even reformulated, whenever new data become available. The present results (Fig. 9a) indicated that the retrained ANNs had predictive capability beyond the explicit training range. Although no experimental data (unavailable) for input power or surface temperature in the 200 V & 5 MPa and 50 V & 5 MPa cases were used to retrain the ANNs, the simulated onsets of flash sintering still matched experimental observations. This suggested that the ANNs provided a degree of robustness outside the training envelope. Nevertheless, such extrapolation is expected to be reliable only within moderate extensions of the operating domain; for more significant departures, prediction accuracy may deteriorate. In practical deployment, on-line retraining would therefore be governed by performance monitoring, with updates triggered only when deviations from experimental measurements exceed a prescribed tolerance, ensuring both adaptability and stability of the digital twin.

An additional consideration for practical deployment is the handling of sparse or noisy datasets, which are common in experimental studies of multiphysics processes. The two-step training strategy adopted in this work already alleviated data sparsity by pretraining ANNs on synthetic data generated from empirical rules, thereby reducing reliance on extensive experimental datasets. Nevertheless, future implementations could integrate transfer learning and data augmentation (Aazami et al., 2021; Aazami & Saidi-Mehrabad, 2019, 2021; Allali et al., 2022; Azad et al., 2019; Goli et al., 2018; Hamlehvar & Aazami, 2025; Ma et al., 2025; Makui et al., 2016; Saeedi Mehrabad et al., 2017; Zarreh et al., 2024). In transfer learning, pretrained ANNs from one material system or process condition are fine-tuned on a smaller dataset from a related system, effectively leveraging prior knowledge to improve generalisation. Data augmentation techniques, such as adding controlled perturbations or noise to synthetic training data, could further improve the robustness of the nested ANNs against experimental uncertainties. Together, these strategies would enhance the resilience of the framework in real-world applications where comprehensive and noise-free datasets are rarely available.

Although implemented within an FE framework for flash sintering, the proposed approach is not limited to this application. A distinctive advantage of the nested training strategy is that it does not require calculation of the derivatives of measured variables with respect to ANN output variables. Since such derivatives are often problem dependent, avoiding them makes the approach broadly generalisable. This opens the possibility of applying the framework to other computational analyses that rely on empirical rules. For example, in computational fluid dynamics, turbulence closures or drag correlations could be replaced with retrainable ANNs updated with experimental flow data, enabling adaptation to specific operating conditions and improving predictive accuracy in complex flow regimes. Likewise, in structural mechanics, empirical constitutive laws for plasticity or creep could be replaced with nested ANNs trained on measured global responses, allowing the model to continuously refine its representation of material behaviour as new data become available. This generality positions the framework as a flexible and powerful tool for developing adaptive, self-improving digital twins across diverse engineering disciplines.

## Conclusions

This study introduced a generalisable framework for nesting and training ANNs within a multiphysics FE analysis, demonstrated through the case of flash sintering. A new backpropagation algorithm integrated with the Adam optimiser was developed to enable the retraining of ANNs while nested within a commercial FE package, thereby transforming conventional FE analysis into a digital twin capable of continual adaptation to experimental data. Replacing empirical rules for electrical conductivity, Joule heating and the constitutive law with retrainable ANNs significantly improved predictive accuracy and reducing strain prediction errors by an order of magnitude compared with conventional models. Validation against the experimental data (Francis & Raj, 2012) confirmed that the ANN-nested framework reliably captured flash onset, input power, surface temperature, deformation trends and energy dissipation, thereby addressing major shortcomings of previous constitutive-equation-based simulations. Scientifically, the results reinforced that rapid temperature rate, rather than absolute temperature, governs flash densification, in agreement with prior experimental studies, and further demonstrated the critical role of contact resistance in energy dissipation, a factor often neglected in earlier modelling. Taken together, these contributions showed that the ANN-nested FE framework not only fulfilled the dual objectives of methodological feasibility and practical applicability but also offered a transferable pathway toward adaptive digital twins for complex manufacturing processes beyond flash sintering.

## Figures and Tables

**Fig. 12:**
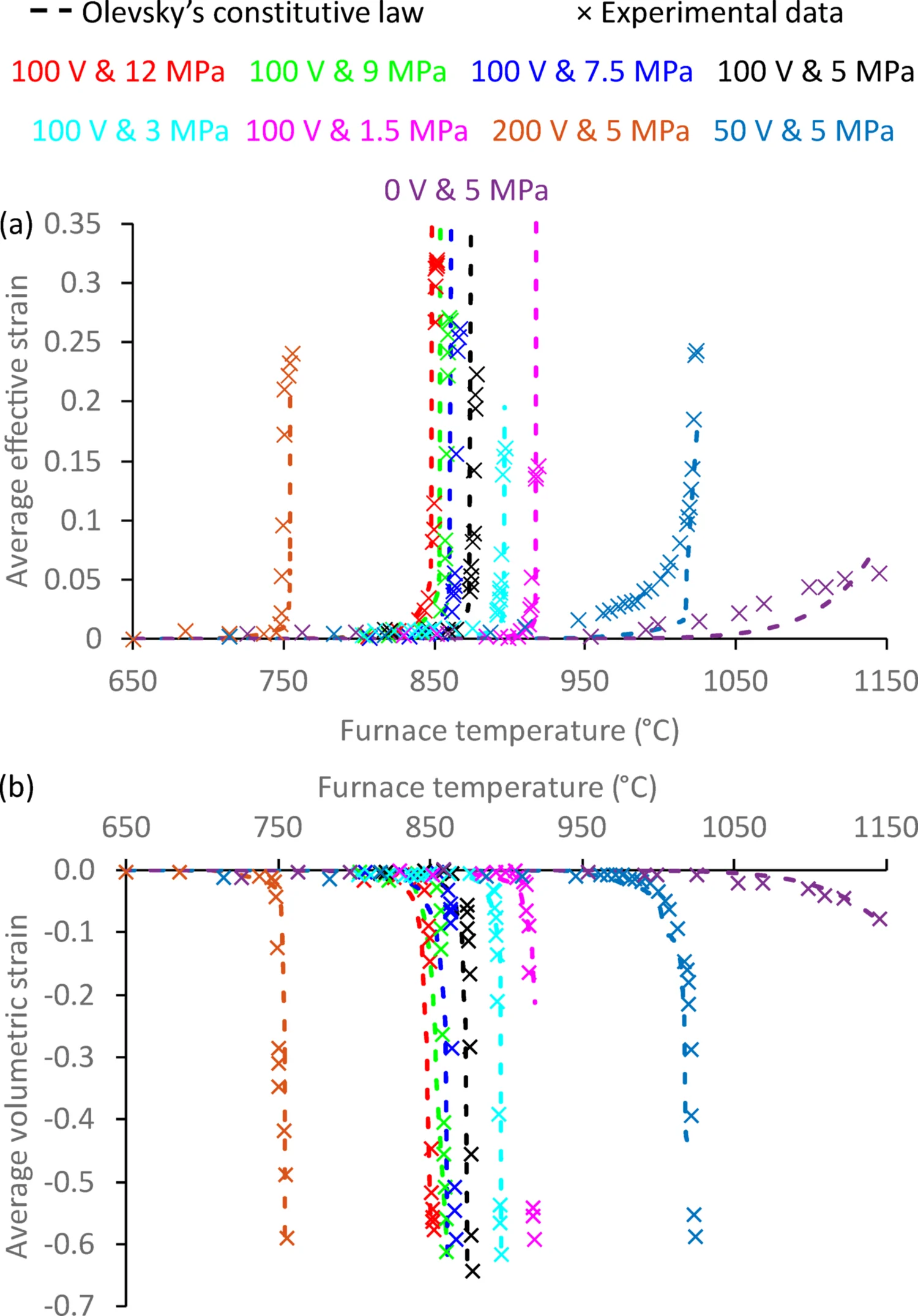
Temporal evolutions of average effective (**a**) and volumetric (**b**) strains, simulated using Olevsky’s constitutive law, in comparison with experimental data (Francis & Raj, 2012)

## Appendix – Olevsky’s constitutive law

The inelastic effective and volumetric strain rates for Olevsky’s constitutive law are expressed as (Van Nguyen et al., 2016).

A1 $$\:\begin{array}{c}{\dot{\varepsilon}}_{e}=\frac{{\sigma\:}_{e}}{3{G}_{p}},\end{array}$$

A2 $$\:\begin{array}{c}{\dot{\varepsilon}}_{v}=\frac{{\sigma\:}_{m}-{\sigma\:}_{s}}{{K}_{p}},\end{array}.$$

where $\:{\sigma\:}_{e}$ is the von Mises stress and $\:{\sigma\:}_{m}$ is the mean stress. The effective shear and bulk viscosities can be described as

A3 $$\:\begin{array}{c}{G}_{p}=\eta\:{{\rho\:}^{r}}^{2n-1},\end{array}$$

A4 $$\:\begin{array}{c}{K}_{p}=3{{f}_{s}}^{2}\eta\:{{\rho\:}^{r}}^{2n-1},\end{array}.$$

where $\:{\rho\:}^{r}$ is the relative density, equal to $\:\rho\:/{\rho\:}_{f}$, and * n* is the material constant, with

A5 $$\:\begin{array}{c}\eta\:={d}^{3}{C}_{s1}T\mathrm{exp}\left(\frac{{C}_{s2}}{RT}\right),\end{array}$$

A6 $$\:\begin{array}{c}{f}_{s}=\frac{1}{2.5\sqrt{1-{\rho\:}^{r}}},\end{array}.$$

where * d* is the grain size, $\:{C}_{s1}$ is the material constant and $\:{C}_{s2}$ is the activation energy for densification. The sintering stress is a function of relative density and grain size, expressed as

A7 $$\:\begin{array}{c}{\sigma\:}_{s}=\frac{4\gamma\:}{\xi\:d}{{\rho\:}^{r}}^{{N}_{s}}{\left[\frac{{\rho\:}^{r}\left(1-{\rho\:}_{g}^{r}\right)}{{\rho\:}_{g}^{r}\left(1-{\rho\:}^{r}\right)}\right]}^{\frac{1}{3}},\end{array}.$$

where $\:\gamma\:$ is the specific surface energy, $\:\xi\:$ is the correction factor for sintering stress and $\:{N}_{s}$ is the exponential fitting parameter.

For the calibration of Olevsky’s constitutive law, the values for * n*, * d*, $\:{C}_{s2}$, $\:\gamma\:$, $\:\xi\:$, $\:{N}_{s}$ and $\:{\rho\:}_{g}^{r}$ remained the same for all cases as listed in Table 5. However, different values of $\:{C}_{s1}$ were obtained from the fittings for different cases (Table 7) and the fitted evolutions of effective and volumetric strains are plotted in Fig. 12. Apparently, the fitted Olevsky’s constitutive law had difficulty in matching the occurrences of rapid changes in the strains and the final strains. Observation of the values in Table 7 revealed a pattern - $\:{C}_{s1}$ increased as the applied voltage or pressure decreased. The major difference across the cases was the temperature rate, which increased with an increase in the applied voltage or pressure. Therefore, it could be concluded that $\:{C}_{s1}$ was temperature rate-dependent for flash sintering. This finding motivated the inclusion of temperature rate as an input variable for the ANN replacing constitutive law. On the other hand, the varying fitted constitutive relationships across different cases made it challenging for the ANN to learn effectively. Under the same relative density, stresses, temperature and temperature rate, different constitutive relationships resulted in different strain rates, which, however, should be the same in reality. To address this, $\:{C}_{s1}$ was modified to be a function of temperature rate, as described in the Mathematical equations for flash sintering, allowing for only one constitutive relationship to be fitted across all cases.

**Table 7:** Values of $\:{C}_{s1}$ in Olevsky’s constitutive law for all cases

Case	$\:{C}_{s1}$ (Pa s)
100 V & 12 MPa	1.15 × 10^7^
100 V & 9 MPa	1.46 × 10^7^
100 V & 7.5 MPa	2.55 × 10^7^
100 V & 5 MPa	3.64 × 10^7^
100 V & 3 MPa	1.09 × 10^8^
100 V & 1.5 MPa	2.91 × 10^8^
200 V & 5 MPa	7.28 × 10^4^
50 V & 5 MPa	7.65 × 10^9^
0 V & 5 MPa	9.14 × 10^10^
